# Effect of different targeted calyxes on the treatment of multi-site calculi in the postural drainage lithotripsy system

**DOI:** 10.3389/fmed.2022.1050118

**Published:** 2023-02-16

**Authors:** Tao Yang, Chong Wu, Liangliang Dai, Zhixiong Hu, Rijin Song, Xianghu Meng, Honglei Shi

**Affiliations:** ^1^Wujin Hospital Affiliated Jiangsu University, Changzhou, China; ^2^The Wujin Clinical College of Xuzhou Medical University, Changzhou, China; ^3^The Affiliated Changzhou Second People's Hospital of Nanjing Medical University, Changzhou Second People's Hospital, Changzhou Medical Center, Nanjing Medical University, Changzhou, China; ^4^The Second Affiliated Hospital of Zhengzhou University, Zhengzhou, China; ^5^Department of Urology, The First Affiliated Hospital of Nanjing Medical University, Nanjing, China

**Keywords:** residual fragment, active lithecbole, postural drainage, urolithiasis, individualized therapy

## Abstract

**Objectives:**

We developed a postural drainage lithotripsy system (PDLS) that can provide an individualized inversion and overturning angle and uses gravity to remove residual fragments (RFs). This study aimed to evaluate the effect of different targeted calyces on treating multi-site stones in PDLS.

**Methods:**

A total of 20 stones with different sizes and diameters of 0–4 mm were placed in the kidney model through ureteroscopy, and 20 stones were evenly scattered in the middle calyx and the lower calyx of the model. The ventral-middle calyx, the dorsal-middle calyx, the ventral-lower calyx, and the dorsal-lower calyx were used as the targeted calyx of PDLS to treat multi-site stones. During treatment, if the stone moved from the starting position of the renal calyx to the ureteropelvic junction, it was recorded as “passing through.” The clearance rate was recorded, and the efficacy of different targeted calyxes in the treatment of multiple-site calyx was compared. Each model was treated with four different targeted calyxes, and 20 models were tested 80 times.

**Results:**

When the lower calyx was the targeted calyx, the total stone clearance rate was higher than when the middle calyx was the locating calyx (94.5 vs. 64%, *P* = 0.000), and the result was statistically significant.

**Conclusions:**

Choosing the lower calyx as the targeted calyx, we can obtain a better stone clearance rate. However, there is no significant difference between the ventral lower calyx and the dorsal lower calyx.

## Introduction

Retrograde internal surgery (RIRS), extracorporeal shock wave lithotripsy (ESWL), and percutaneous nephrolithotomy (PCNL) are the main methods for the treatment of urinary calculi (1). However, whatever treatment method is adopted, residual stones (RFs) will inevitably occur (2), especially RIRS (3). Within 2 years after minimally invasive surgery, 26% of the patients with RFs will have symptoms such as renal colic, obstruction, infection, and stone recurrence and thus need reintervention (4, 5). In addition, repeated surgery or other treatment will reduce patients' quality of life, and additional medical care costs will be incurred (6, 7). Therefore, urologists should pay attention to the complete clearance of RFs.

In our previous research, we proposed new equipment and technology, the postural drainage lithotripsy system (PDLS), which can make the patient's body reach a specific and accurate spatial position to use gravity to promote the spontaneous removal of residual stones (8). This study initially aimed at treating residual stones in a single renal calyx, but clinically, residual stones are numerous and distributed in all corners of the kidney after an operation. In addition, we designed an *in vitro* trial to evaluate the effect of PDLS on treating multi-site stones.

## Materials and methods

### Postural drainage lithotripsy system

**Figure 1:**
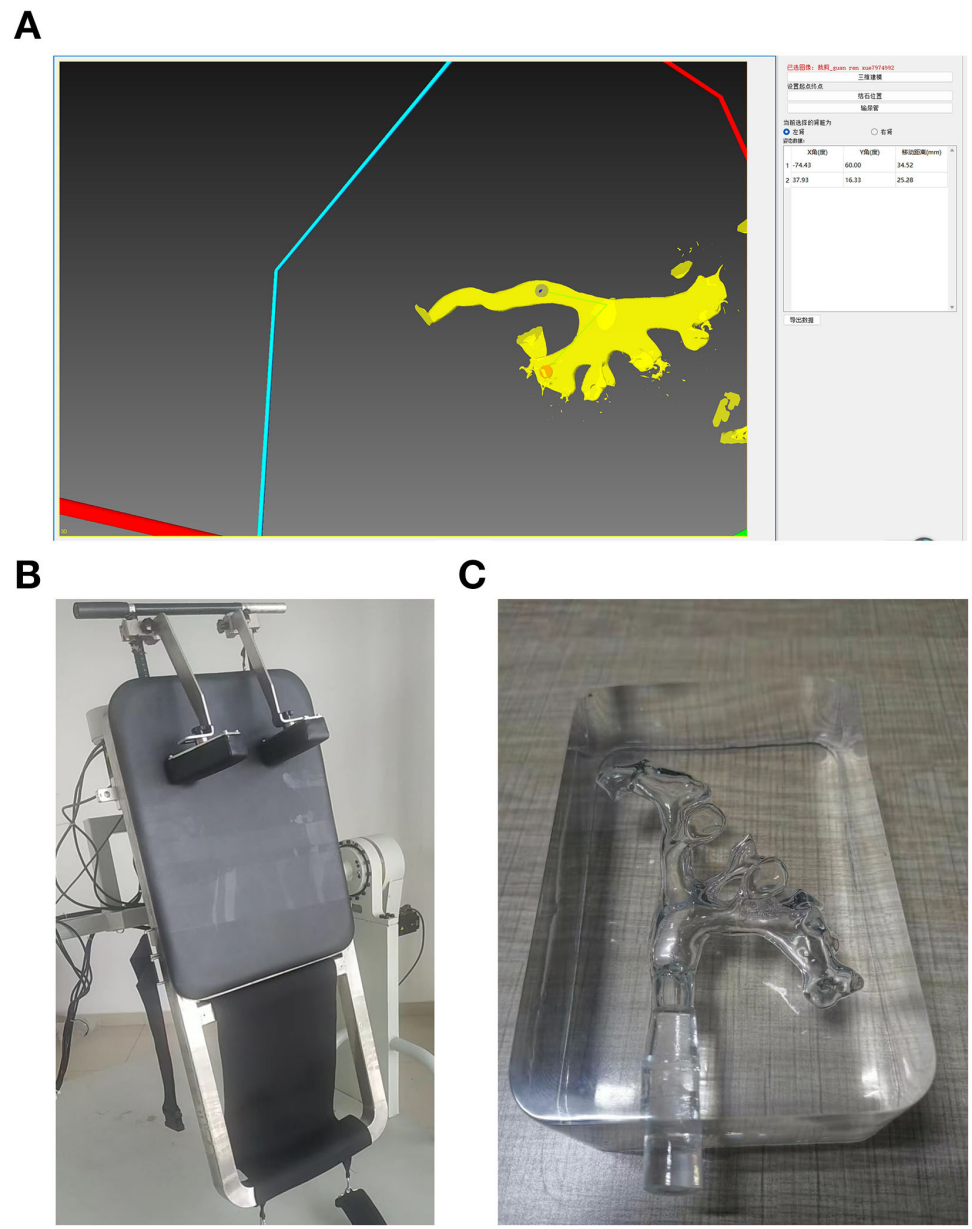
Postural drainage lithotripsy system. **(A)** Stone clearance path planning software, the red point is the targeted calyx, and the blue point is in the UPJ. (−74, 60) and (37, 16) are the (*X, Y*)-axis parameters of the space rotating bed. **(B)** The space rotating bed. **(C)** 3D printed kidney models.

Our previous study showed that PLDS could accurately remove residual stones in the kidney (8). The PDLS includes stone clearance path planning software and a space-rotating bed, which uses the patient's computed tomography urography (CTU) data to form a visual image of the 3D kidney collection system (8). After locating the stone location and calculating and providing personalized inversion and turnover angles, the rotating space bed will achieve a specific space posture to use gravity to promote the spontaneous excretion of residual stones (Figure 1).

### 3D-printed kidney models

CTU data from patients were used to create 20 transparent silicone renal pelvis models using 3D printing technology. The ureter and the bladder were cleaned during the modification, and the urine was sealed in the kidney model. The kidney's small kidney stones were sent to the renal calyx through the flexible ureteroscope.

### Multi-site stones

**Figure 2:**
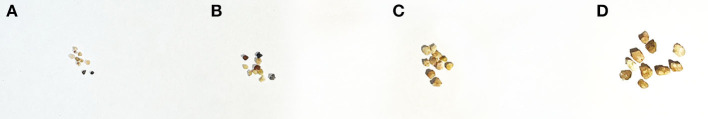
Stone used in the experiment (diameter range 1–4 mm). **(A)** 0–1 mm, **(B)** 1–2 mm, **(C)** 2–3 mm, **(D)** 3–4 mm.

Small stones were divided into four groups according to their maximum diameter: A, B, C, and D. The diameter of group A was 0–1 mm; the diameter of Group B was 1–2 mm; the diameter of Group C was 2–3 mm; and the diameter of Group D was 3–4 mm. Five stones were taken from each group, with a total of 20 stones in Group E. The stones in Group E were placed in 20 kidney models through ureteroscopy, and the stones in Group E were evenly scattered in the middle and lower calyxes of the model (Figure 2).

### Procedures

The definition of “targeted calyx” refers to when there are multiple-site stones. We selected one of the renal calyxes as the stone location and set it as the starting point of PDLS. The renal calyx is called a targeted calyx. As the stones were scattered in the middle and lower calyxes of the kidney, the targeted calyxes were divided into four groups: the ventral-middle calyx (VM), the dorsal-middle calyx (DM), the ventral-lower calyx (VL), and the dorsal-lower calyx (DL).

The doctor imported the CTU image data from the model through the stone clearance path planning software and reconstructed the 3D renal pelvis model, locating the four renal calyxes (VM, DM, VL, and DL) on the corresponding 3D model, respectively. The endpoint of the stone was set at the ureteropelvic junction (UPJ). The software calculated the cleaned path of the stone and converted it into the X and Y-axis parameters of the space-rotating bed and then input the specified rotation parameters on the control panel of the space-rotating bed. Each model was treated with four different targeted calyces, and 20 models were tested 80 times.

### Observation items

If the stone moved from the initial position of the renal calyx to the UPJ during the treatment, it was recorded as “passing through.”

### Statistical methods

SPSS 26.0 software was used for the statistical analysis of the data. The median and quartile were used to express the number of middle and lower calyxes and the total number of stones cleaned. The rank-sum test was used to compare the components. The result was statistically significant (*P* < 0.05).

## Results

Table 1 shows that, when the lower calyx was the targeted calyx, the total stone clearance rate was higher than when the middle calyx was the locating calyx (94.5 vs. 64%, *P* = 0.000), and the result was statistically significant. For the same dorsal-ventral calyx, the clearance rate of the lower calyx was higher than that of the middle calyx (93.75 vs. 62.75%, *P* = 0.000). For the same ventral calyx, the clearance rate of the lower calyx was also higher than that of the middle calyx (95.25 vs. 65.25%, *P* = 0.000).

**Table 1:** Stone clearance rate of four targeted calyces.

**Targeted calyx**	**Number of stones cleaned**		
	**Total**	**Middle calyx**	**Lower calyx**
DM	251/400 (62.75%)	193/200 (96.5%)	58/200 (29%)
VM	261/400 (65.25%)	197/200 (98.5%)	64/200 (32%)
Middle calyx total	512/800 (64%)	390/400 (97.5%)	122/400 (30.5%)
DL	375/400 (93.75%)	195/200 (97.5%)	180/200 (90%)
VL	381/400 (95.25%)	198/200 (99%)	183/200 (91.5%)
Lower calyx total	756/800 (94.5%)	393/400 (98.25%)	363/400 (90.75%)

### Middle calyx

When the middle calyx was used as the targeted calyx, the total stone clearance rate was 512 out of 800 (64%), the number of middle calyx stones cleaned was 390 out of 400 (97.5%), and the number of lower calyx stones cleaned was 122 out of 400 (30.5%; Table 1).

The clearance rates of VM and DM were similar (65.25 vs. 62.75%, *P* = 0.982), and the result was not statistically significant. In the DM group, the number of stones cleaned in the middle calyx was 193 out of 200 (96.5%), and the number of stones cleaned in the lower calyx was 58 out of 200 (29%). In the VM group, 197 out of 200 (98.5%) stones were cleaned from the middle calyx and 64 out of 200 (32%) stones were cleaned from the lower calyx (Tables 1, 2).

**Table 2:** Number of stones cleaned when the middle calyx (VM and the DM) is the targeted calyx.

	**Number of stones cleaned**		***P*-value**
	**VM**	**DM**	
Middle calyx	10 (10, 10)^a^	10 (10, 10)	0.102
Lower calyx	3.5 (1.25, 5)	4 (1, 5.75)	0.867
Total	13.5 (11.25, 15)	14 (11, 15.75)	0.982

a 10 (10, 10) means median (interquartile range) in Tables 2, 3.

### Lower calyx

When the lower calyx was used as the targeted calyx, the total stone clearance rate was 756 out of 800 (94.5%), the number of middle calyx stones cleaned was 393 out of 400 (98.25%), and the number of lower calyx stones cleaned was 363 out of 400 (90.75%; Table 1).

The clearance rates of VL and DL were also similar (95.25 vs. 93.75%, *P* = 0.974), and the result was not statistically significant. In the DL group, the number of stones cleaned in the middle calyx was 195 out of 200 (97.5%), and the number of stones cleaned in the lower calyx was 180 out of 200 (90%). In the VL group, 198 out of 200 (99%) stones were cleaned from the middle calyx, and 183 out of 200 (91.5%) stones were cleaned from the lower calyx (Tables 1, 3).

**Table 3:** Number of stones cleaned when the lower calyx (VL and the DL) was the targeted calyx.

	**The number of stones cleaned**		***P*-value**
	**VL**	**DL**	
Middle calyx	10 (10, 10)	10 (9.25, 10)	0.180
Lower calyx	9 (8, 10)	10 (8, 10)	0.811
Total	19 (18, 20)	19.5 (18, 20)	0.974

## Discussion

Doctors have gradually become concerned about the treatment of RFs after minimally invasive surgery for urinary calculi. These RFs may cause complications such as steinstrasse, renal colic, obstruction, infection, and stone recurrence (9). Some scholars studied the use of gravity to treat residual stones with an inverted posture; however, these methods have limited effects and cannot achieve accurate treatment (10). The PDLS system we developed succeeded in the preliminary study, and a single residual stone in the kidney model was cleaned properly (8). In the clinic, urinary calculi are usually crushed by the energy of the Holmium laser in the kidney and moved to different renal calyxes. Therefore, these stone powders are traditionally difficult to remove. In the PDLS, the doctor can only select one stone location in the clearance path planning software and then select the endpoint. Thus, choosing one of the many calyxes as the best-targeted calyx for treating multi-site stones has become the main objective of this study.

This experiment's results showed that the lower calyx's stone clearance rate was much higher than that of the middle calyx (94.5 vs. 64%, *P* = 0.000). Therefore, we can conclude that the lower calyx can be used as the locating calyx to get a better clearance rate when treating multi-site RFs. We believe that this result can guide the treatment of clinical patients in future work.

At the same time, we also noticed that, when the middle calyx was used as the targeted calyx, the clearance rate of the VM group had only a slight advantage over the DM group (65.25 vs. 62.75%, *P* = 0.982). However, statistically, this advantage was not significant. Similar results were also found in the lower calyx. The stone clearance rate of the VL group was higher than that of the DL group (95.25 vs. 93.75%, *P* = 0.974). This suggests that, although the stone clearance rate is higher when the lower calyx is located, there is no significant difference between the DL and VL groups (Tables 1, 3).

When the middle calyx was used as the targeted calyx, the total stone clearance rate was 512 out of 800 (64%), the number of middle calyx stones cleaned was 390 out of 400 (97.5%), and the number of lower calyx stones cleaned was 122 out of 400 (30.5%). This indicates that, when the middle calyx is used as the targeted calyx for treatment, PDLS can remove a large number of stones located in the middle calyx but only a small number of stones located in the lower calyx.

When the lower calyx was used as the targeted calyx, the total stone clearance rate was 756 out of 800 (94.5%), the number of middle calyx stones cleaned was 393 out of 400 (98.25%), and the number of lower calyx stones cleaned was 363 out of 400 (90.75%). This suggests that, when the lower calyx is used as the targeted calyx for treatment, PDLS has a good effect on both the stones of the middle calyx and the lower calyx. Although both VL and DL belong to the lower calyx, the clearance rates of VL and DL are also similar (95.25 vs. 93.75%, P = 0.974), and the result was not statistically significant.

Whether the stone can be cleaned depends on the diameter of the stone and the diameter of the calyx and the ureters. The diameter of the stone used in this experiment is 1–4 mm, and the diameter of the normal renal calyx is also >4 mm. Because the stones are small enough to pass through the calyx and the ureters, there is no need to discuss the clearance rate of stones of different sizes. However, we will further study the significance of this parameter in future clinical studies.

We acknowledge that there are limitations to the present study. First, we did not systematically study the shape of stones because there are many shapes after the operation, and the existing experimental conditions are insufficient to support this plan. Additionally, we only confirmed the above results from *in vitro* experiments; they have not been verified in clinical experiments. However, we still believe they can guide future clinical experiments.

## Conclusions

Our present study shows that choosing the lower calyx as the targeted calyx can obtain a better stone clearance rate; however, there is no significant difference between the DL and VL. When the lower calyx is used as the targeted calyx for treatment, PDLS has a good effect on both the stones of the middle calyx and the lower calyx. PDLS provides a new idea for the individualized therapy of residual stones, and we will conduct further clinical trials to verify its therapeutic effects.

## Data availability statement

The raw data supporting the conclusions of this article will be made available by the authors, without undue reservation.

## Ethics statement

Written informed consent was obtained from the individual(s) for the publication of any potentially identifiable images or data included in this article.

## Author contributions

TY and CW: project development and manuscript writing. LD: data collection and data analysis. ZH: visual processing of experimental results. RS: experimental design supervision and leadership. XM: review and revision of the first draft. HS: manuscript and digital writing/editing. All authors significantly contributed to the findings and methods in this study, read, and approved the final draft.
